# Breakthrough Curve Modeling and Analysis for Lysozyme Adsorption by Tris(hydroxymethyl)aminomethane Affinity Nanofiber Membrane

**DOI:** 10.3390/membranes13090761

**Published:** 2023-08-28

**Authors:** Kuei-Hsiang Chen, You-Ren Lai, Nguyen The Duc Hanh, Steven S.-S. Wang, Yu-Kaung Chang

**Affiliations:** 1 Department of Chemical Engineering, Graduate School of Biochemical Engineering, Ming Chi University of Technology, New Taipei City 243303, Taiwan; 2 Department of Chemical Engineering, National Taiwan University, Taipei 10617, Taiwan; 3 Department of Chemical Engineering and Materials Science, Yuan Ze University, Zhongli Dist., Taoyuan City 320315, Taiwan

**Keywords:** membrane bed chromatography, tris(hydroxymethyl)aminomethane affinity nanofiber membrane, lysozyme, dynamic kinetic studies, breakthrough curve models

## Abstract

In this study, a polyacrylonitrile nanofiber membrane was first hydrolyzed and then functionalized with tris(hydroxymethyl)aminomethane (P-Tris), then used as an affinity nanofiber membrane for lysozyme adsorption in membrane chromatography. The dynamic adsorption behavior of lysozyme was investigated in a flow system under various operating parameters, including adsorption pHs, initial feed lysozyme concentration, loading flow rate, and the number of stacked membrane layers. Four different kinetic models, pseudo-first-order, pseudo-second-order, Elovich, and intraparticle diffusion kinetic models, were applied to experimental data from breakthrough curves of lysozyme. The results showed that the dynamic adsorption results were fitted well with the pseudo-second-order kinetic model. The breakthrough curve experimental results show significant differences in the breakthrough time, the dynamic binding capacity, the length of the mass transfer zone, and the utilization rate of the membrane bed under different operating parameters. Four dynamic adsorption models (i.e., Bohart–Adams, Thomas, Yoon–Nelson, and BDST models) were used to analyze the breakthrough curve characteristics of the dynamic adsorption experiments. Among them, the Yoon–Nelson model was the best model to fit the breakthrough curve. However, some of the theoretical results based on the Thomas and Bohart–Adams model analyses of the breakthrough curve fit well with the experimental data, with an error percentage of <5%. The Bohart–Adams model has the largest difference from the experimental results; hence it is not suitable for breakthrough curve analysis. These results significantly impact dynamic kinetics studies and breakthrough curve characteristic analysis in membrane bed chromatography.

## 1. Introduction

Nanofiber membranes are widely used in protein adsorption and separation [1,2,3,4,5,6,7,8], wastewater treatment [9,10,11,12,13], and pharmaceutical and biomedicine applications [14,15,16,17,18]. Previous work found tris(hydroxymethyl)aminomethane affinity nanofiber membranes could be successfully applied to lysozyme recovery from chicken egg white in continuous flow systems [1,2,4,8]. Compared with traditional resin chromatography, nanofiber membrane chromatography has the advantages of a low pressure drop, high flux, and high binding capacity. Although batch reactors can be easily operated on the laboratory scale, the batch mode is not suitable for practical use. Membrane adsorption chromatography has been confirmed to be an effective process in continuous flow systems due to its simplicity and ease of operation and processing [1,2,4,8,19,20,21]. Breakthrough curves are important features for obtaining dynamic binding characteristics [1,2,4,5,8]. Either the shape or slope of the breakthrough curve is commonly used to assess the dynamic binding performance of a membrane absorber in a flow system.

In previous work [9], various cation-exchange nanofiber membranes, such as weak ion-exchange nanofiber membranes (i.e., P-COOH), strong ion-exchange nanofiber membranes (i.e., P-SO_3_H), and amphoteric ion-exchange nanofiber membranes (i.e., P-COOH-BSA; BSA, namely, bovine serum albumin) were synthesized via hydrolysis and coupling reactions. The dynamic binding performance for divalent metal ions (i.e., Ca^2+^) and breakthrough of these nanofiber membranes were evaluated at different loading concentrations, flow rates, and stacked membrane layers. Two mathematical models (i.e., Thomas and bed depth service time) were used to predict the breakthrough curve. The results indicated that Ca^2+^ removal by these nanofiber membranes was very effective due to the high interface mass transfer. The Thomas model was more suitable for elucidating the experimental result trends observed from the dynamic adsorption system. Until now, a limited amount of literature has reported the adsorption behavior of lysozyme in nanofiber membrane flow systems [8]. To our knowledge, there is no related work on the use of tris affinity nanofiber membranes to fit the breakthrough curve of lysozyme using a mathematical model.

The purpose of this work was to use the designed nanofiber membrane reactor to determine the dynamic binding performance of lysozyme in a flow system. The effects of process parameters such as adsorption pH, inlet lysozyme concentration, liquid flow rate, and the number of stacked membrane layers on the breakthrough characteristics of the adsorption system were investigated. In this work, various dynamic adsorption models, such as the Bohart–Adams model, Thomas model, Yoon–Nelson model, and bed depth service time (BDST) model [22,23,24,25,26,27,28] were used to evaluate and predict the adsorption performance of the tris(hydroxymethyl)aminomethane affinity nanofiber membrane (namely, P-Tris) for lysozyme. The mathematical models used to study the breakthrough characteristics are described in Section 2.6. To analyze the breakthrough curve characteristics of the dynamic binding experiments, important performance indicators calculated from the breakthrough curves were used to assess the dynamic binding performance of the P-Tris nanofiber membrane to lysozyme (e.g., dynamic binding capacity (*DBC*), maximum (equilibrium) binding capacity (*EBC*), mass transfer zone length (HMTZ), membrane adsorbent exhaustion rate (*MAER*), membrane bed utilization (*MBU*, %), and productivity of lysozyme (*P*) [9].

Furthermore, adsorption kinetics provide valuable information on the possible adsorption mechanisms and their potential rate-limiting step during the process. Batch kinetic adsorption experiments are carried out by adding a known amount of adsorber (e.g., one piece of membrane, 0.03 g) to a fixed liquid solution (e.g., 5 mL) at a known initial lysozyme concentration (e.g., 2 mg/mL) and a fixed agitation rate (e.g., 150 rpm), and recording the evolution in time of the lysozyme concentration [9]. In this work, the dynamic kinetic adsorptions in the membrane beds were carried out in flow systems. The different adsorption behaviors for lysozyme adsorption processes in the batch reactor and the membrane flow reactor may be caused by the controlling mechanisms in each operating system. In the adsorption processes that use a membrane bed column, the mechanism of intraparticle mass transfer is due to axial and radial dispersion. However, this mechanism would not happen in the batch reactor. Four different kinetic models, pseudo-first-order, pseudo-second-order, Elovich, and intraparticle diffusion kinetic models, were applied to experimental data from the breakthrough curves of lysozyme [7,8,9].

According to the difference between the model calculation results and the experimental data, the applicability of the model to describe the dynamic adsorption model of membrane adsorbent to lysozyme can be evaluated. The linear regression coefficient (R^2^) and percent error (E, %) are used to assess the fit correlation of the model to the breakthrough curve of the experimental results and to verify the consistency of the adsorption model predictions.

## 2. Materials and Methods

### 2.1. Materials and Apparatus

All materials and apparatus were obtained previously from published work [1,9,10]. PAN membrane (molecular weight 120,000 g/mol) was obtained from Fortune Industries Inc. (New Taipei City, Taiwan). Polyethylene terephthalate (PET) nonwoven fabric was procured from Freudenberg Far Eastern Spunweb Co., Ltd. (Taoyuan City, Taiwan) [19]. The electrospinning device was obtained from Falco Tech Enterprise Co., Ltd. (New Taipei City, Taiwan) [19]. A purpose-designed membrane holder (25 mm internal diameter) was used in the dynamic adsorption experiments [19]. Lysozyme (EC 3.2.1.17) and other chemicals were purchased from Sigma-Aldrich (St. Louis, MO, USA).

### 2.2. Preparation of Tris(hydroxymethyl)aminomethane Affinity Nanofiber Membrane

For the preparation of tris(hydroxymethyl)aminomethane affinity nanofiber membrane, refer to our previous article [8]. The analyses of physical properties of the tris(hydroxymethyl)aminomethane affinity nanofiber membrane was performed by using scanning electron microscopy (SEM; Hitachi S-2600H, Tokyo, Japan), Fourier transform infrared (FTIR) spectroscopy (Perkin Elmer Spectrum One), and thermogravimetric analysis (TGA; Mettler Toledo, Q600, Columbia, MD, USA).

### 2.3. Membrane Flow Systems

A three-layer structure of the affinity nanofiber membrane (~0.03 g) was used in the experiment. It consisted of ~0.015 g of polyethylene terephthalate and ~0.015 g of upper and lower double-layer affinity nanofiber membranes. An AKTA Prime system (GE Healthcare Biosciences, Uppsala, Sweden) was used with a membrane reactor system (membrane effective area of 3.7 cm^2^) as described in previous work [9,10,14,19]. Experiments were performed with various operating parameters including adsorption pH (i.e., 5, 7, and 9), lysozyme concentration (i.e., 0.5, 1.0, and 2.0 mg/mL), loading flow rate (i.e., 0.1, 0.5, and 1.0 mL/min), and membrane stack layers (i.e., 1, 3, and 5), and their effect on the dynamic binding of lysozyme was assessed. To calculate the inlet and outlet concentrations of lysozyme, dynamic breakthrough curves were made. The lysozyme content in the liquid phase was determined using UV–vis spectroscopy (GE Healthcare Biosciences, Sweden) [19]. The residence time (Equation (1)) and permeation flux (Equation (2)) in the membrane holders can also be calculated [9,19].

The permeation flux, *J* (L/m^2^·h·bar) in the flow system was calculated using Equation (1) [9,20]:

(1) $$J=\left(\frac{V}{A\times t}\right)$$

The residence time in the membrane, *τ* (min) was determined using Equation (2) [9,21]:

(2) $$\tau =\frac{\varepsilon \times V_{M}}{F}$$

where *V_M_* represents the membrane volume (~3.39 × 10^−2^ mL), *F* represents the flow rate (0.1–1.0 mL/min), and ε represents the porosity of the membrane (~83.67%).

### 2.4. Breakthrough Analysis

In the dynamic experiment, when the concentration of lysozyme at the outlet is equal to the concentration at the inlet (i.e., *C/C_o_* = 1), total binding capacity of the nanofiber membrane obtained from the breakthrough curve is correspondent to that of the equilibrium dynamic binding capacity (*EBC*) of the nanofiber membranes [9]. In this work, the dynamic binding capacity (*DBC*) was calculated at the 10% breakthrough point [9], where the volume and duration time at the breakthrough point are expressed as *V_b_* (mL) and *t_b_* (i.e., 10% breakthrough time), respectively [9,19]. 

The breakthrough time (*t_b_*), defined as the time at which the normalized lysozyme concentration (*C/C_o_*) was equal to 0.1, was determined from the breakthrough curves by non-linear regression analysis.

The productivity (P) is calculated by using Equation (3) [28]:

(3) $$P=\left(\frac{DBC}{t_{b}}\right)$$

Membrane bed utilization (*MBU*, %) is defined as the ratio of the *DBC* to the *EBC* using Equation (4) [28]:

(4) $$MBU(\%)=\left(\frac{DBC}{EBC}\right)\times 100$$

In addition, the bed volume (*BV*) is represented as the volume ratio of the feed volume at which lysozyme concentration is at 10% breakthrough (*V_b_*) to the membrane adsorber bed volume (*V_M_*). The dynamic binding performance can be represented as the membrane adsorber exhaustion rate (*MAER*) [9,19] of the membrane bed employed throughout the experiment. The *MAER* is the membrane mass required (g) per unit of lysozyme adsorbed at 10% of the breakthrough (mL). Furthermore, the length of the mass transfer zone (*HMTZ*) is the adsorption region of the lysozyme that occurs in the membrane bed, where the concentration of lysozyme at the outlet reaches between 10% and 90% of the concentration at the inlet, and this is the region where most of the mass transfer occurs [9,19]. The adsorption efficiency of the membrane bed is dependent on the length of the mass transfer zone. If the mass transfer has a thinner zone, it results in a higher adsorption performance of the membrane bed [9].

### 2.5. Kinetic Rate Constant

Plots of *q_t_* versus *t* are called dynamic kinetic curves. The dynamic kinetic curve can be easily converted from *C/C_o_* versus *t* breakthrough curves [9]. The dynamic kinetic models selected depend on an appropriate mathematical relation used to represent the kinetic curve [10]. To model the dynamic kinetic results in flow systems, batch kinetic models were also used in dynamic kinetic models [19].

The dynamic binding capacity (*q_t_*) of the nanofiber membrane bed was calculated using Equation (5):

(5) $$q_{t}=\frac{F\times \int_{0}^{t}\left(C_{0}-C_{t}\right)dt}{W}$$

where *F* is the flow rate (mL/min), *C*_0_ is the feedstock concentrations of lysozyme, and *C_t_* (namely, *C*) is the outlet concentration from the column bed at time (*t*) (mg/mL); *W* is the mass of the nanofiber membrane, g; and t is the time in min. 

The dynamic experiment data obtained from the breakthrough were fitted into different kinetic models to investigate the controlling mechanism of adsorption and the rate constant of adsorption. The experiment data were fitted with four kinetic models. Adsorption on the nanofiber was described by pseudo-first-order (Equation (6)) and pseudo-second-order (Equation (7)), Elovich (Equation (8)), and intraparticle diffusion (Equation (9)) kinetic models [10,19]. The linearized kinetic models are as follows:

(6) $$ln\left(q_{1}-q_{t}\right)=lnq_{1}-{\;k}_{1}t$$

(7) $$\left(\frac{t}{q_{t}}\right)=\left(\frac{1}{k_{2}q_{2}^{2}}\right)-\left(\frac{1}{q_{2}}\right)t$$

(8) $$q_{t}=\frac{1}{\beta }ln\left(\alpha \beta \right)+\frac{1}{\beta }ln\left(t\right)$$

(9) $$q_{t}=k_{i}\times t^{0.5}+I$$

where *k_i_* is the intraparticle diffusion rate constant, mg/g·min^0.5^. *k_i_* value was determined from the slopes of straight-line portions of the respective plots; *q*_1_, *q*_2_, and *q_t_* represent the binding capacity (mg/g) of the dye at any given time, t; and at equilibrium, *t* is the adsorption time; *k*_1_ and *k*_2_ are the pseudo-first-order and pseudo-second-order kinetics rate constants, respectively. α and β are the Elovich kinetic constants.

### 2.6. Breakthrough Curve Models

Four breakthrough curve models including Bohart–Adams, Thomas, Yoon–Nelson, and BDST models were applied to the analysis of the breakthrough curve parameters [22,23,24,25,26,27]. The modeling parameters can be determined from the slope and intercept obtained from the linear relationship of each adsorption model.

#### 2.6.1. Bohart–Adams Model

In the Bohart–Adams model [29], the binding rate is proportional to the residual binding capacity of the membrane adsorber bed and concentration of the adsorbed lysozyme. The linear expression is given based on Equation (10):

(10) $$ln\left(\frac{C_{t}}{C_{o}}\right)=\left(k_{BA}C_{o}t-\frac{k_{BA}N_{o}Z}{v}\right)$$

The *k_BA_* and *N_o_* are determined from the slope and intercept of the plot of ln (*C_t_*/*C_o_*) against time at a given membrane bed depth and liquid linear velocity.

#### 2.6.2. Thomas Model

The Thomas model is based on a Langmuir-type adsorption process with second-order reversible kinetics [30]. The adsorption rate is not controlled by the external or internal mass transfer, but by the interface mass transfer. This model is commonly used to evaluate the relationship between concentration and time in breakthrough curves, as shown in Equation (11):

(11) $$ln\left(\frac{C_{o}}{C_{t}}-1\right)=\left(\frac{k_{T}\cdot q_{e}\cdot W_{M}}{F}\right)-\left(k_{T}\cdot C_{o}\cdot t\right)$$

The *k_T_* and *q_e_* can be determined from the slope and intercept, respectively, of the plot of ln(*C_o_*/*C_t_* − 1) against time at a given flow rate.

#### 2.6.3. Yoon–Nelson Model

The Yoon–Nelson model was used to determine the probability of each lysozyme molecule being adsorbed. This is proportional to its adsorption and breakthrough probability on the membrane adsorber [31]. A linear expression is given by Equation (12):

(12) $$ln\left(\frac{C_{t}}{{C_{o}-C}_{t}}\right)=\left(k_{YN}t-k_{YN}\tau \right)$$

The *k_YN_* and *t*_0.5_ are determined from the slope and intercept of the plot of ln (*C_t_*/(*C_o_* − *C_t_*)) against time, respectively.

#### 2.6.4. BDST Model 

Bohart and Adams proposed the BDST model [29], in which the determination of the adsorption rate is controlled by a surface reaction of the lysozyme and the unused binding capacity. The BDST model demonstrated a linear relationship between the service time and the column bed height, as shown in Equation (13) [32].

(13) $$t=\left(\frac{Q_{o}\cdot Z}{C_{o}\cdot v}\right)-\left(\frac{1}{k_{BDST\cdot }C_{o}}\right)\times ln\left(\frac{C_{o}}{C_{t}}-1\right)$$

where *k_BDST_* (mL/mg·min) is the kinetic constant, and *a* (slope) and *b* (intercept) are given, respectively, by:

(14) $$a=\left(\frac{Q_{o}\cdot Z}{C_{o}\cdot v}\right)$$

(15) $$b=\left(\frac{1}{k_{BDST}\cdot C_{o}}\right)\times ln\left(\frac{C_{o}}{C_{t}}-1\right)$$

The BDST analysis is useful for adsorption process design in the scale-up and is applied to predict the breakthrough time of membrane bed systems at 10% and 90% breakthrough.

#### 2.6.5. Linear Regression Coefficients and Error Analysis

Linear regression coefficients and error analysis can be used to assess the confidence of the model in describing the adsorption performance of the breakthrough curve. In general, the linear regression coefficient (*R^2^*) indicates how close the breakthrough curve parameters obtained from the linearized adsorption equation (see Equations (1) to (4)) are to the experimental results. The error percentage (*E*, %) indicates the degree of difference between the experimental results and the theoretical values [9].

## 3. Results and Discussion

### 3.1. Process Parameters

**Figure 1:**
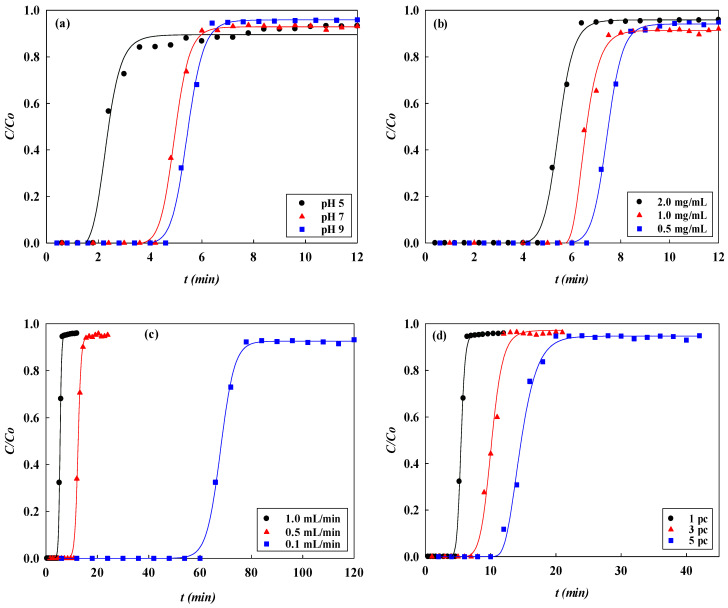
The breakthrough curves of P-Tris nanofiber membrane for lysozyme adsorption at different (**a**) pHs, (**b**) lysozyme concentrations, (**c**) flow rates, and (**d**) numbers of stacked membrane layers.

In breakthrough curve experiments, different process parameters including pH, inlet concentration of lysozyme, loading flow rate, and the number of membrane layers can affect the dynamic binding performance of the membrane adsorber. Various effects of the process parameters on the dynamic adsorption performance are shown in Figure 1.

#### 3.1.1. Effect of Adsorption pH

**Figure 2:**
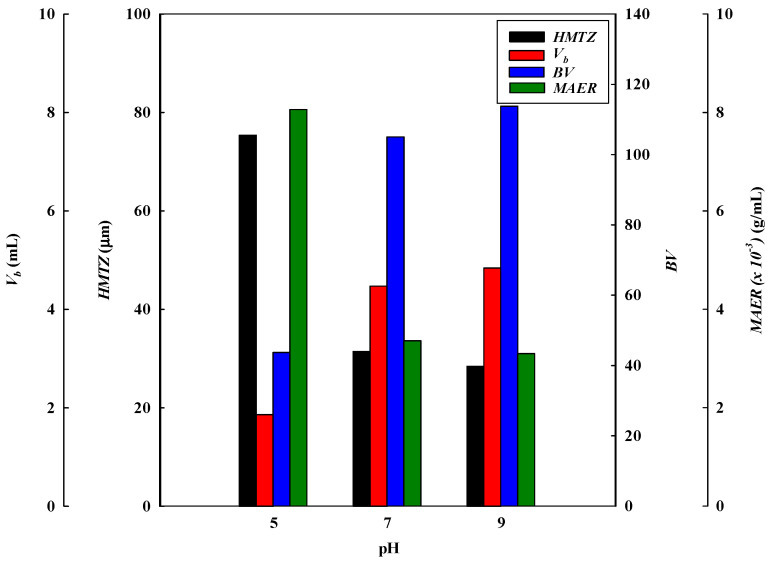
Breakthrough curve analysis for *V_10%_*, *BV*, *MAER*, and *HMTZ* at different pH values.

**Figure 3:**
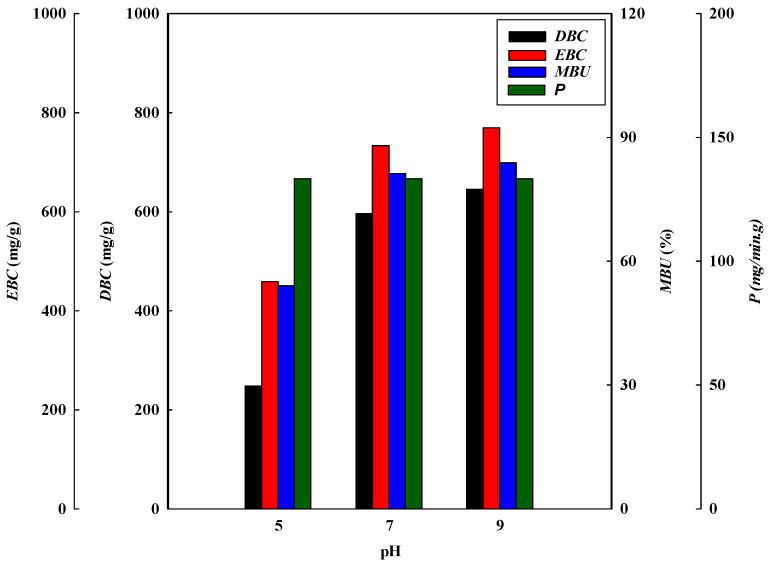
Breakthrough curve analysis for *DBC*, *EBC*, *MBU*, and *P* at different pH values.

The effect of the adsorption pH on the dynamic binding performance of the P-Tris nanofiber membrane (one layer) was studied by varying the pH from 5 to 9 at a constant flow rate of 1 mL/min of 2.0 mg/mL of lysozyme. Figure 1a shows the comparison of the *V_b_* and BV and the breakthrough curve analysis results are shown in Figure 2 and Figure 3. It was found that the values of *V_b_* and *BV* increased with the increasing pH value. Hence, the calculated *DBC* at 10% breakthrough for lysozyme was in decreasing order of pH, i.e., pH 9 (645.33 mg/g) > pH 7 (596.00 mg/g) > pH 5 (248.00 mg/g). The calculated *EBC* for lysozyme was observed at pH 5, 7, and 9, with adsorption capacities of 459.05 mg/g and 735.55 mg/g, and 769.65 mg/g, respectively. On the contrary, the values of the MAER and *HMTZ* decreased with the increase in pH value, and the lower values of these parameters indicated a higher binding performance of the P-Tris membrane. Hence, the optimal binding for lysozyme at pH 9 was chosen for the subsequent operating condition.

The results of the dynamic binding performance in the pH range selected may be due to the lower surface charge on the P-Tris nanofiber membrane. This may be due to a reduction in the effective charge–charge interaction between lysozyme and the P-Tris membrane at a lower, acidic pH value of 5, thus leading to a decrease in the binding capacity of the membrane. Similar findings were also observed elsewhere [9,19]. In addition, the values of *MBU* increased with increasing pH value, however, the productivity (*P*) of lysozyme at the breakthrough point at these pH values is similar. The *MBU* values for pH 5, pH 7, and pH 9 are 54.03%, 81.03%, and 83.85%, respectively. However, the productivity for these adsorption pHs has a similar value of 133.33 mg/min·g.

#### 3.1.2. Effect of Feedstock Lysozyme Concentration

The effect of the loading lysozyme concentrations at pH 9 on the binding performance of the P-Tris membrane (one layer) at 1 mL/min of flow rate and pH 9 is presented in Figure 1b. The results indicate that the loading lysozyme level can affect the breakthrough curve to a significant extent. It is obvious that the calculated breakthrough parameters were remarkably affected by the concentration of lysozyme loaded onto the system. An early breakthrough point from the membrane bed was observed when a higher concentration of lysozyme was used.

**Figure 4:**
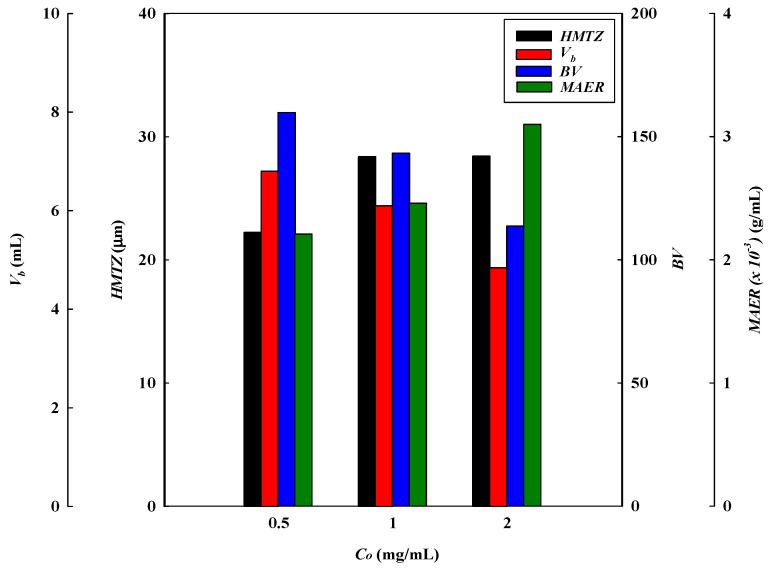
Breakthrough curve analysis for *V_10%_, BV, MAER,* and *HMTZ* at different feed concentrations of lysozyme.

**Figure 5:**
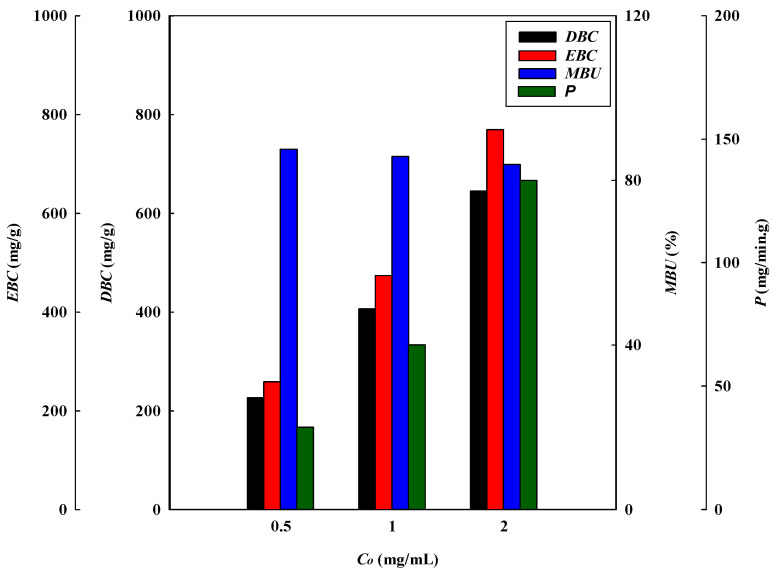
Breakthrough curve analysis for *DBC, EBC, MBU,* and *P* at different feed concentrations of lysozyme.

The breakthrough curve analysis results are shown in Figure 4 and Figure 5. It was found that the values of *V_b_* and *BV* decreased with an increasing lysozyme concentration in the feedstock. At a higher lysozyme concentration, the binding sites on the membrane adsorber were more rapidly filled with lysozyme molecules, resulting in a shorter breakthrough time. Therefore, the lysozyme volume loaded at breakthrough point (*V_b_*) was found to decrease from 6.80 to 4.84 mL (corresponding to BV decreasing from 159.81 to 113.75) as the lysozyme concentration increased from 0.5 to 2.0 mg/mL. The volume processed at the breakthrough point decreased with an increase in lysozyme concentration, while the *DBC* increased from 226.67 mg/g to 769.65 mg/g when the lysozyme concentration increased from 0.5 to 2.0 mg/mL. Similarly, the *EBC* increased from 258.75 mg/g to 769.65 mg/g. The gradient of the lysozyme concentration acted as a driving force for mass transfer, where a higher lysozyme level led to a greater mass transfer of lysozyme to the membrane adsorber surface. This phenomenon led to a higher dynamic binding capacity for lysozyme.

The calculated *MAER* was found to increase from 2.21 × 10^−3^ g/mL to 3.10 × 10^−3^ g/mL when increasing the lysozyme concentration from 0.5 to 2.0 mg/mL. In the adsorption process, the driving force for the adsorption of lysozyme increases with increasing lysozyme concentration. Consequently, the binding sites have a higher saturation when subjected to a higher concentration of lysozyme, and this led to a higher adsorber exhaustion rate. In this case, the values of *HMTZ* increased from 22.24 μm to 28.43 μm as the lysozyme concentration increased from 0.5 to 2.0 mg/mL. This indicates that a higher concentration of lysozyme may lower the binding performance for lysozyme. The membrane adsorber utilization (*MBU*) decreased from 87.60% to 83.85% as the lysozyme concentration increased from 0.5 to 2.0 mg/mL. On the contrary, the productivity (P) increased from 33.33 to 133.33 (mg/min·g).

#### 3.1.3. Effect of Flow Rate

**Figure 6:**
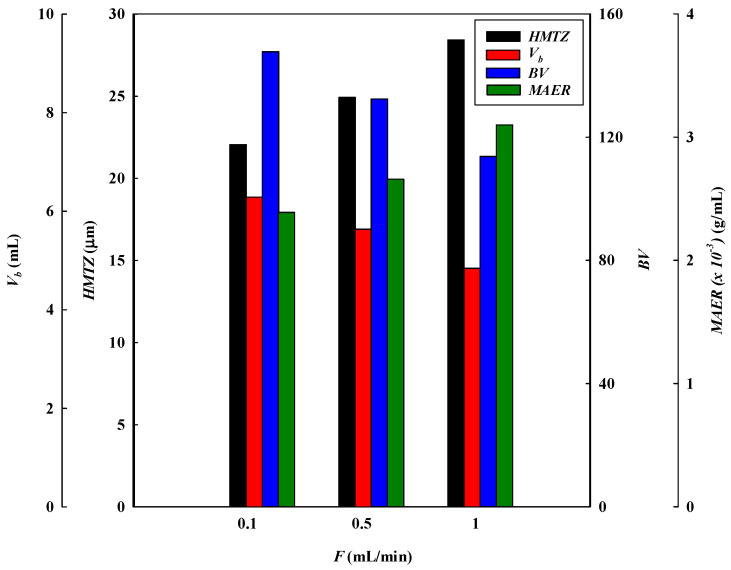
Breakthrough curve analysis for *V_10%_, BV, MAER,* and *HMTZ* at different loading flow rates.

**Figure 7:**
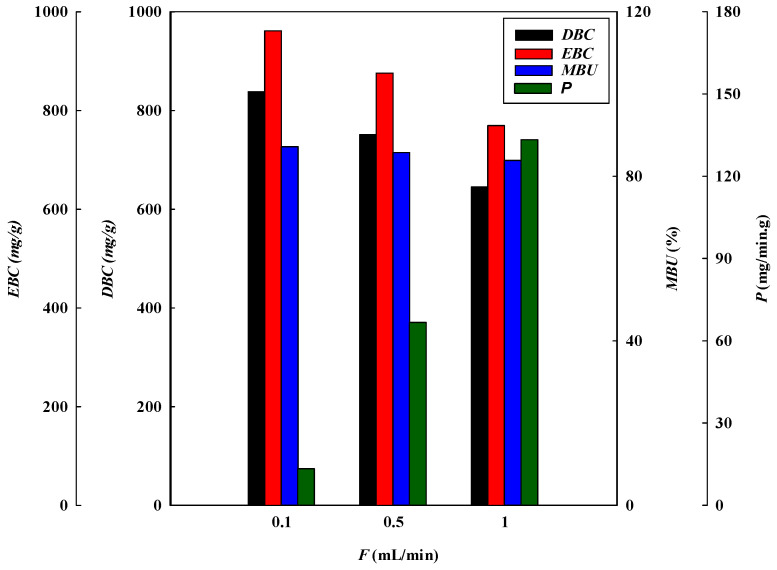
Breakthrough curve analysis for *DBC*, *EBC*, *MBU*, and *P* at different loading flow rates.

The loading flow rate is also an important parameter in designing membrane chromatography. The breakthrough curves at different flow rates (i.e., 0.1, 0.5, and 1.0 mL/min) at a lysozyme concentration of 2.0 mg/L, pH 9, and with a one-layer P-Tris membrane are depicted in Figure 1c. The breakthrough curve analysis results are shown in Figure 6 and Figure 7. These indicate that the time required to reach the 10% breakthrough point decreases when the flow rate increases. Specifically, as the flow rate increased from 0.1 mL/min to 1.0 mL/min, the time required to reach the breakthrough point decreased from 62.87 to 4.28 min. The decrease in breakthrough time with an increase in flow rate may be due to the decrease in the residence time in the membrane bed. The volume loaded at the breakthrough point (*V_b_*) and the bed volume (*BV*) of lysozyme decreased from 6.29 to 4.84 mL and from 147.76 to 113.75, respectively, with an increase in the flow rate from 0.1 to 1.0 mL/min.

The *DBC* and *EBC* values decreased from 838.27 to 645.33 mg/g and 964.59 to 875.87 mg/g, respectively, as the flow rate increased from 0.1 to 1.0 mL/min. In this case, the residence time (*τ*) in the membrane bed was not long enough for the capture of lysozyme. Therefore, as the contact time between lysozyme and the membrane bed is shorter at a higher flow rate, there is a reduction in the dynamic binding capacity.

Additionally, the *MAER* and *HMTZ* values were observed to increase from 2.39 × 10^−3^ to 3.10 × 10^−3^ g/mL and 20.06 to 28.43 μm, respectively, when the flow rate increased from 0.1 to 1.0 mL/min. In the adsorption process, a higher flow rate led to a higher *MAER* value, representing a lower binding performance (Table 1). This is due to a shorter mass transfer zone (*HMTZ*) for the capture of lysozyme on the membrane bed [9,19]. The productivity of lysozyme increased from 13.33 to 133.33 mg/min·g when increasing the flow rate from 0.1 mL/min to 1.0 mL/min. Therefore, a higher flow rate is beneficial for increasing productivity (*P*). The *MBU* of lysozyme decreased from 86.90 to 83.85 mg/min·g when increasing the flow rate from 0.1 mL/min to 1.0 mL/min.

#### 3.1.4. Effect of Stacked Membrane Layers

**Figure 8:**
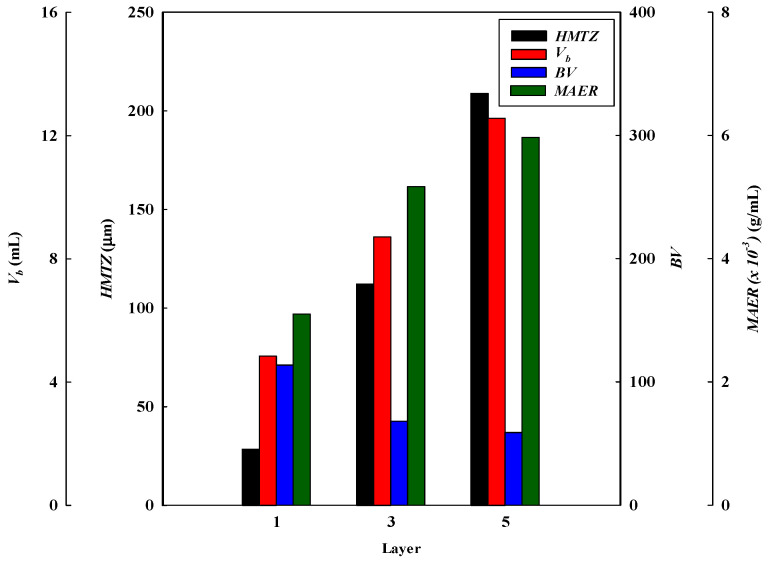
Breakthrough curve analysis for *V_10%_*, *BV*, MAER, and *HMTZ* at different stacked membrane layers.

**Figure 9:**
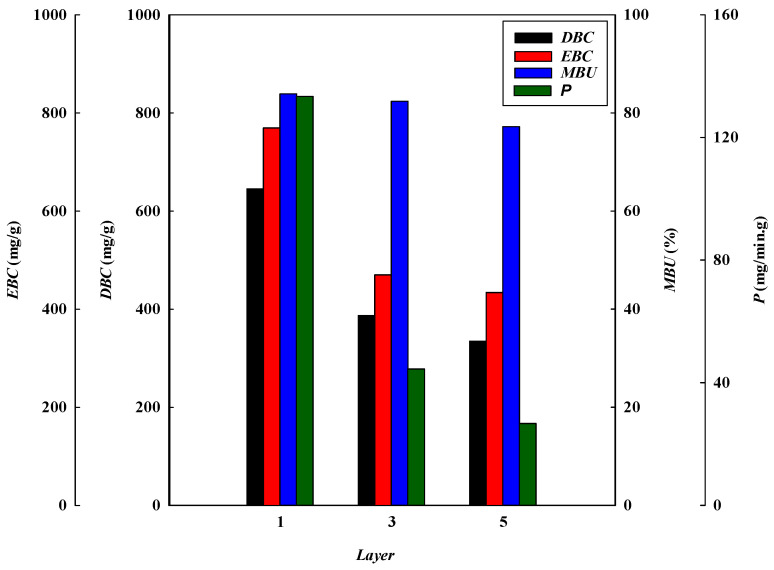
Breakthrough curve analysis for *DBC*, *EBC*, *MBU*, and *P* at different stacked membrane layers.

The number of stacked membrane layers is also an important process parameter affecting the dynamic binding performance. It determines the number of available binding sites for the adsorption of lysozyme. Experiments were carried out by increasing the membrane layers from one to five under an optimized condition (i.e., lysozyme concentration of 2.0 mg/L at pH 9 and a flow rate of 1 mL/min). Figure 1d describes the breakthrough curves obtained at different layers of the membrane bed. The breakthrough curve analysis results are shown in Figure 8 and Figure 9. It was observed that the breakthrough point was highly dependent on the membrane layers. An earlier breakthrough point was observed when subjected to one layer of the membrane in the bed and there was a sharper rise in the effluent lysozyme concentration. This may be due to the presence of a shorter mass transfer zone in the membrane bed. It is observed that the breakthrough time (*t_b_*) increased with increasing the layers of the membrane, in which there were more binding sites present for lysozyme in a multi-layer membrane. In these cases, the volumes of lysozyme loaded at the breakthrough point (*V_b_*) were 4.84, 8.71, and 12.56 mL, with the total volumes of the membrane beds corresponding to 0.04255, 0.12765, and 0.21275 mL, respectively. 

Additionally, the *DBC* values decreased from 645.33 to 349.93 mg/g when increasing the layers of the membrane from one to five. The *DBC* for lysozyme in the multi-layer membrane bed at 10% breakthrough was not fast enough to effectively capture the lysozyme. However, the *EBC* values increased from 769.65 to 433.79 mg/g. The increased *EBC* with an increase in the number of membrane layers can be explained by the fact that lysozyme molecules have a longer contact time and more available binding sites when there are more layers of membranes. Hence, at the final equilibrium stage, the *EBC* value increased when increasing the number of membrane layers.

Meanwhile, the calculated *MEAR* and *HMTZ* values increased from 3.10 × 10^−3^ to 5.97 × 10^−3^ g/mL and 28.43 to 208.96 μm with an increase in the number of layers of the membrane bed from one (115 μm) to three (575 μm). The results show that a higher value of the *MAER* and *HMTZ*, lowers the binding performance of lysozyme in the membrane bed. The productivity of lysozyme and *MBU* (%) decreased from 133.33 to 27.86 mg/min·g and 83.85% to 80.67%, respectively, when increasing the number of membrane layers from one to five. Therefore, it is advantageous for increasing the productivity of lysozyme to use a one-layer membrane adsorber. Similar results are also observed in membrane bed utilization.

A comparison of the breakthrough curve analysis and parameters obtained from different process conditions is shown in Table 1. Table 1 shows an increase in binding with the addition of more layers. The addition of a second layer increases the total binding capacity (mg) by about 79.96%, whereas a third layer improves the total binding capacity (mg) by an additional 159.50%. Hence, the total binding capacity increased with an increasing number of stacked layers. The total binding capacity was determined at 10% breakthrough in these cases. The reason is that the one-layer membrane has a lower number of binding sites than two- and three-layer membranes. During the adsorption stages, these flow systems were not blocked, and the liquid permeate flux (160.04 L/m^2^·h·bar) was not dramatically affected. However, it was found that the binding capacity for lysozyme per gram of membrane (mg/g) decreased as the number of membrane layers increased. For one-layer, two-layer, and three-layer membranes, the binding capacities for lysozyme by the P-Tris nanofiber membrane were 645.33 mg/g, 387.11 mg/g, and 334.93 mg/g, respectively. The reason for this phenomenon is that the porosity distribution of the entire film is not uniform, and the liquid flow cannot allow the solution to flow uniformly to the whole membrane in the second and third layers after the first layer is attached by the lysozyme molecules.

### 3.2. Dynamic Kinetic Studies

**Figure 10:**
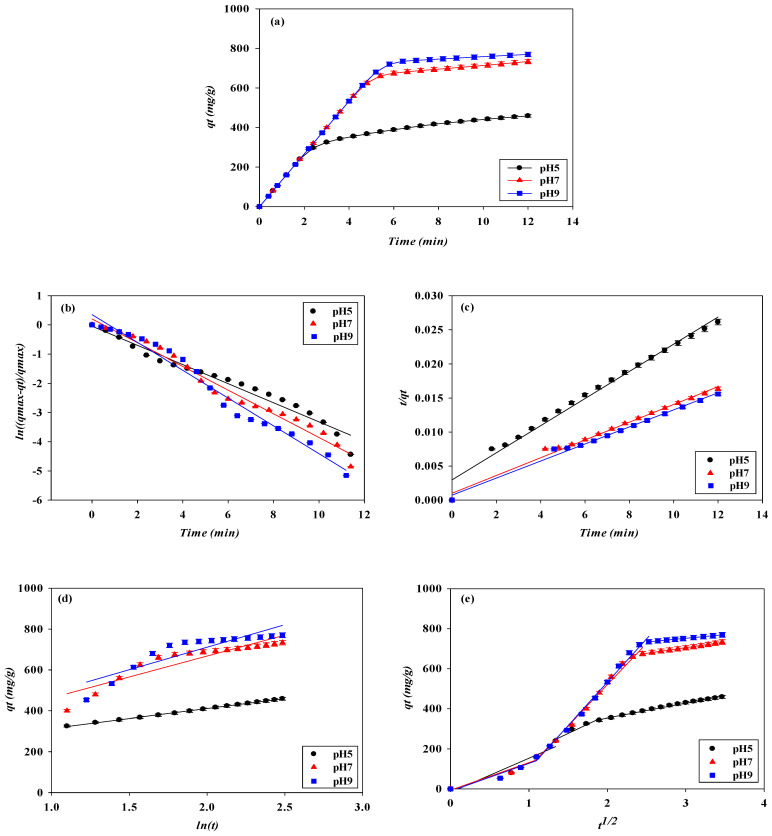
(**a**) Adsorption rates for lysozyme by P-Tris nanofiber membrane under different pH values. Kinetic adsorption of lysozyme fitted by (**b**) pseudo-first-order model, (**c**) pseudo-second-order model, (**d**) Elovich, and (**e**) Intraparticle diffusion model.

**Figure 11:**
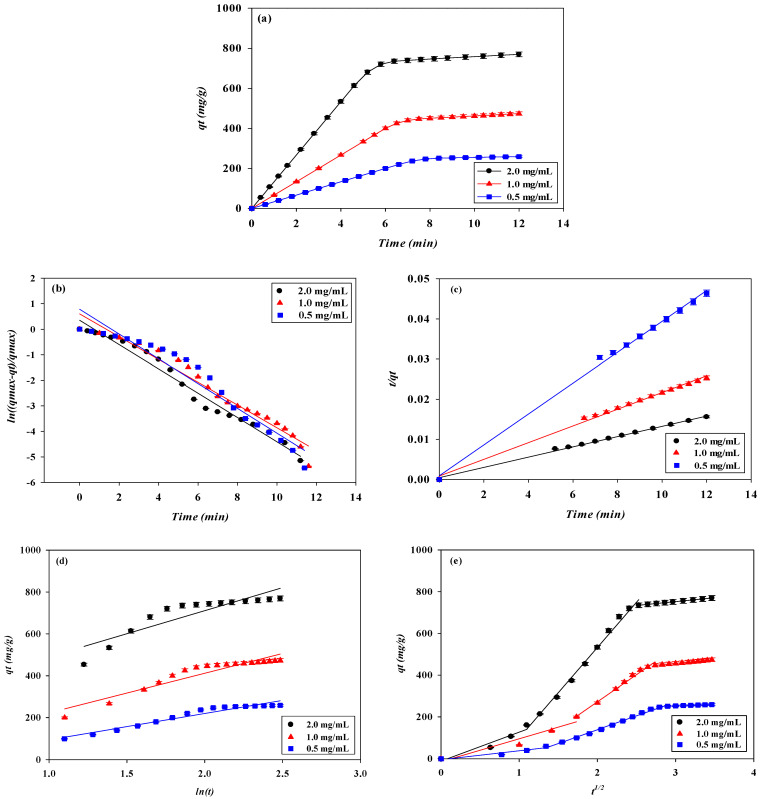
(**a**) Adsorption rates for lysozyme by P-Tris nanofiber membrane under different lysozyme concentrations. Kinetic adsorption of lysozyme fitted by (**b**) pseudo first-order model, (**c**) pseudo-second-order model, (**d**) Elovich, and (**e**) intraparticle diffusion model.

**Figure 12:**
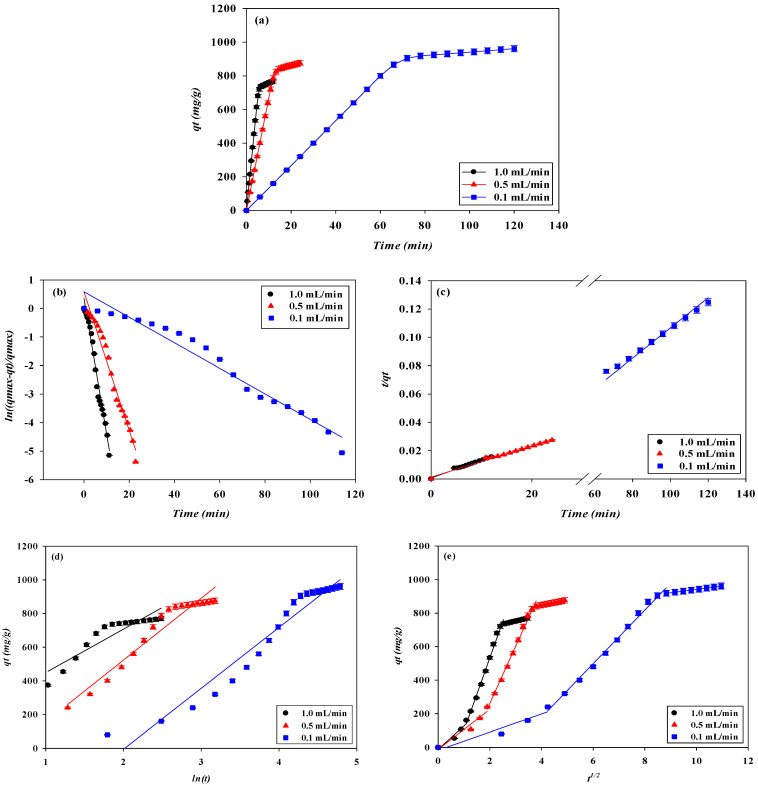
(**a**) Adsorption rates for lysozyme by P-Tris nanofiber membrane under different flow rates. Kinetic adsorption of lysozyme fitted by (**b**) pseudo first-order model, (**c**) pseudo-second-order model, (**d**) Elovich, and (**e**) intraparticle diffusion model.

**Figure 13:**
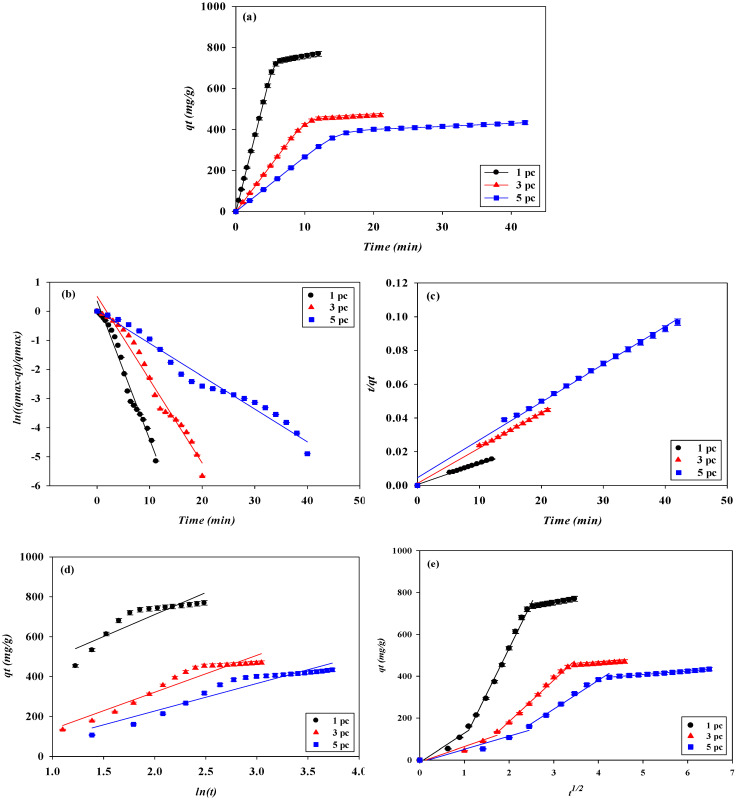
(**a**) Adsorption rates for lysozyme by P-Tris nanofiber membrane under different membrane stacking layers. Kinetic adsorption of lysozyme fitted by (**b**) pseudo-first-order model, (**c**) pseudo-second-order model, (**d**) Elovich, and (**e**) intraparticle diffusion model.

To identify the kinetics that controls the adsorption mechanism of lysozyme on the P-Tris nanofiber membrane under our experimental conditions, pseudo-first-order, pseudo-second-order, Elovich, and intraparticle diffusion kinetic models were applied to the experimental data from the breakthrough curves of lysozyme. Under the four different operating conditions (e.g., adsorption pH, feedstock lysozyme concentration, loading flow rate, and membrane stacking layers), all values of the coefficient of determination (R^2^ > 0.99) calculated using the pseudo-second-order kinetic model were higher than those estimated using the other three kinetic models (e.g., pseudo-first-order, Elovich, and intraparticle diffusion kinetic models), as shown in Figure 10, Figure 11, Figure 12 and Figure 13. 

This shows that the kinetic parameters calculated by the pseudo-second-order model are in good agreement with the experimental results, and the reaction mechanism of the adsorption kinetics of lysozyme on the P-Tris nanofiber membrane indicates that the surface reaction on the membrane may be the rate-determining step which can be used to describe the kinetic mechanism of lysozyme adsorption. Using 0.5, 1.0, and 2.0 mg/mL lysozyme concentrations, the obtained lysozyme adsorption capacity (*q_cal_*) is 260.08, 480.77, and 798.72 mg/g, respectively. It is noteworthy that these values are quite close to the maximum amount of the adsorbed lysozyme (*q_exp_*) by P-Tris as shown in Table 2. The values of the rate constant *k_2_* decrease with increasing initial lysozyme concentration. The reason for this behavior can be attributed to the lower competition for the adsorption surface sites at lower concentrations. At higher concentrations, the competition for the surface binding sites was high and consequently lower adsorption rates were obtained. Similar phenomena have been observed in other adsorption systems [33]. 

To gain an insight into the mechanisms and rate-controlling steps affecting the kinetics of adsorption, the kinetic experimental results were fitted to the Weber’s intraparticle diffusion kinetic model [34]. The kinetic results were analyzed by the intraparticle model to elucidate the diffusion mechanism; the model is expressed as Equation (5). As shown in Figure 11d, it was observed that when the lysozyme concentration is between 0.5 mg/mL and 2.0 mg/mL, the immobilized dye membrane for the adsorption of lysozyme has a three-stage diffusion. If the rate-limiting step is intraparticle diffusion, a plot of adsorption capacity against the square root of the contact time should yield a straight line passing through the origin [35,36,37]. The intraparticle diffusion plots show multi-linearity in the biosorption process, indicating that three steps are operational. The first, sharper stage can be attributed to the diffusion of lysozyme through the solution to the external surface of the membrane or the boundary layer diffusion of the lysozyme. The second stage describes the gradual adsorption, where intraparticle diffusion is rate-limiting, and the third stage is attributed to the final equilibrium for which the intraparticle diffusion starts to slow down due to an extremely low lysozyme concentration left in the solution. The three stages in the plot suggest that the adsorption process occurs by surface adsorption and intraparticle diffusion (microporous). The values of the intraparticle diffusion rate constant, *k_i_*, calculated are shown in Table 2. The results indicate that the intraparticle diffusion rate increases with increasing initial lysozyme concentration in solution. An increase in the initial concentration of lysozyme yields a higher concentration gradient which eventually leads to faster diffusion and rapid adsorption. Furthermore, as the flow rate increased, the combination of the external mass transfer and pore diffusion increased, resulting in the kinetic rate constant increasing.

### 3.3. Modelling of the Breakthrough Curves

In this work, four dynamic adsorption models, namely, Bohart–Adams, Yoon–Nelson, Thomas, and BDST, were used to fit the experimental data and the dynamic parameters were computed using the linearized model equations (see Equations (5)–(8)).

#### 3.3.1. Bohart–Adams model

The values of *No* and *k_BA_* were determined based on the intercept and slope of the Adams–Bohart plot for different process parameters, as shown in Table 3. The *N_0_* value calculated by the Bohart–Adams model increased with pH, lysozyme concentration, and the number of membrane layers. However, the results indicated that the *N_0_* value decreased when subjected to an increase in the flow rate. The calculated parameters of this model did not well fit to the experimental data, and it has the worst fit for *R^2^* values (0.777–0.978) and *E* (%) (13.890–22.326%) of all the models, implying that this model is not suitable for simulating the breakthrough curves in any cases.

#### 3.3.2. Thomas Model

As shown in Table 2, the rate constant (*k_T_*) increased with increasing flow rate, however, it decreased when subjected to a higher concentration of lysozyme and number of membrane layers. A higher concentration, lower flow rate, and multi-layer membranes showed better dynamic binding performance. The breakthrough curves predicted by the Thomas model were compared with the experimental data. There was a large discrepancy between the experimental and predicted results for some of the experiments; while the differences between the values predicted by the Thomas model and some of the experimental results were in the acceptable range (<5%). The fitted regression coefficient (*R^2^*) was between 0.884 and 0.999, and the percentage error (*E* %) was between 1.316% and 36.989%.

#### 3.3.3. BDST Model

The breakthrough parameters fitted by the BDST model are shown in Table 2. The *Q_o_* value increased when increasing the lysozyme concentration and decreased when increasing the feed flow rate or the number of membranes. However, the results indicated that the *k_BDST_* decreased with the increase in the concentration of lysozyme and the number of membranes but increased when subject to a higher flow rate. The results showed that, except for at pH 5, the predicted *Q_o_* values when using the BDST model were quite close to the experimental values. The *R^2^* and *E* (%) values for the BDST model were between 0.884 and 0.999 and between 0.159% and 45.799%, respectively. Therefore, the application of the BDST model in the membrane adsorption process for lysozyme would be acceptable under some operating parameters.

#### 3.3.4. Yoon–Nelson Model

The Yoon–Nelson model was applied to fit the breakthrough curve to obtain satisfactory results, as shown in Table 2, and the Yoon–Nelson rate constant (*k_YN_*) decreased with the increase in the lysozyme concentration and the number of membranes, but *k_YN_* increased with the increase in the flow rate, which is due to more lysozyme molecules passing through the membrane bed. In addition, the value of τ decreased with an increase in the lysozyme concentration, flow rate, and number of membranes. The predicted values obtained from this model were compared with the experimental values, and the simulated results were close to the experimental results as compared to the other models. It was found that the linear regression coefficients (*R^2^*) obtained by the simulations were in the range of 0.884–0.999. The percentage error (*E* %) was in the range of 0.487–7.401%. Therefore, the Yoon–Nelson model was found to be very suitable for representing the adsorption behavior of lysozyme by the P-Tris nanofiber membrane.

### 3.4. Remarks on the Model Fitting 

In assessing the breakthrough curve, the *MAER* and *BV* values at the 10% breakthrough point were defined as the performance indicators. In this work, at a lower lysozyme concentration, higher flow velocity, and higher number of stacked membrane layers, a lower value of *MAER* and a larger value of *BV* were obtained, which indicated a good binding performance. Membrane bed adsorption performance may be strongly influenced by membrane chemical composition and specific features of the porous structure and the membrane surface, including hydrophobic/hydrophilic properties, membrane charge density, surface roughness, pore size, and pore size distribution.

Comparing the simulation results to the experimental data, the order of the degree of how well the breakthrough curves derived using theoretical parameters fit to the experimental data was YN > BDST > Thomas > BA model. Therefore, the breakthrough curve was not well fit by the Bohart–Adams model. However, the breakthrough curve at some process parameters can be well described by the BDST and Thomas models. These models exhibited a lower percentage error (*E* < 5%). Among these models, the application of the Yoon–Nelson model has shown the best agreement with the experimental results; hence, this model can be used for fitting of breakthrough curves.

## 4. Conclusions

The fabricated P-Tris affinity nanofiber membrane was used for the dynamic adsorption of lysozyme by membrane chromatography under various dynamic conditions, such as adsorption pH, feed lysozyme concentration, loading flow rate, and stacked membrane layers. In dynamic kinetic studies, the adsorption behavior for lysozyme was well described by the pseudo-second-order kinetic model. The dynamic binding performance for lysozyme was strongly affected by the operating parameters. At the optimal adsorption pH (pH 9), the dynamic binding capacity (*DBC*) and equilibrium binding capacity (*EBC*) were 645.33 and 769.65 mg/g, respectively. The *DBC* values decreased from 645.33 to 349.93 mg/g when increasing the number of membrane layers from one to five and the EBC values also decreased from 769.65 to 433.79 mg/g. Hence, it is advantageous for increasing the productivity of lysozyme to use a one-layer membrane adsorber. Under the optimal conditions in this work, the maximum values of *BV*, *MBU* (%), and productivity were 159.81, 87.60%, and 133.33 mg/min·g. Moreover, the Yoon–Nelson model was found to describe the breakthrough curve characteristics better than other models. Hence, the Yoon–Nelson model exhibited the best-fit results for the dynamic binding of lysozyme onto the P-Tris affinity membrane. The dynamic adsorption results obtained in this study would apply to various functional nanofiber membranes in flow systems and be helpful for the development of scale-up procedures in membrane bed chromatography.

## Figures and Tables

**Table 1:** Breakthrough curve analysis and parameters obtained from different process conditions.

Process Parameters				Breakthrough Curve Analysis									
*Z* (μm)	pH	*C_o_* (mg/mL)	*F* (mL/min)	*t_0.1_*	*t_0.9_*	*HMTZ*	*V_b_*	*BV*	*MAER* (×10^−3^)	*DBC*	*EBC*	*MBU* (%)	Productivity at DBC (mg/min·g)
115	5	2.0	1.0	1.86	5.40	75.39	1.86	43.71	8.06	248.01	459.05	54.03	133.33
115	7	2.0	1.0	4.47	6.15	31.42	4.47	105.05	3.36	596.01	733.55	81.25	133.33
115	9	2.0	1.0	4.84	6.43	28.43	4.84	113.75	3.10	645.33	769.65	83.85	133.33
115	9	0.5	1.0	6.80	8.43	22.24	6.80	159.81	2.21	226.67	258.75	87.60	33.33
115	9	1.0	1.0	6.10	8.10	28.39	6.10	143.36	2.46	406.67	473.96	85.80	66.67
115	9	2.0	1.0	4.84	6.43	28.43	4.84	113.75	3.10	645.33	769.65	83.85	133.33
115	9	2.0	0.1	62.87	77.79	22.06	6.29	147.76	2.39	838.27	961.59	87.18	13.33
115	9	2.0	0.5	11.27	14.39	24.93	5.64	132.43	2.66	751.33	875.87	85.78	66.67
115	9	2.0	1.0	4.84	6.43	28.43	4.84	113.75	3.10	645.33	769.65	83.85	133.33
115	9	2.0	1.0	4.84	6.43	28.43	4.84	113.75	3.10	645.33	769.65	83.85	133.33
345	9	2.0	1.0	8.71	12.91	112.24	8.71	68.23	5.17	387.11	469.92	82.38	44.44
575	9	2.0	1.0	12.56	19.73	208.96	12.56	59.04	5.97	334.93	433.79	77.21	26.67

**Table 2:** Kinetic analysis for the adsorption of lysozyme by using various models under different operating conditions.

Kinetic Models	pH			Lysozyme (mg/mL)			Flow Rate (mL/min)			Stacked Membrane Layers		
	5	7	9	0.5	1	2	0.1	0.5	1	1	3	5
*q_e,exp_* (mg/g)	459.05	733.55	769.65	258.75	473.96	769.65	961.59	875.87	769.65	769.65	469.92	433.79
Pseudo-first-order												
*k_1_*	0.3269	0.4055	0.4758	0.4853	0.4457	0.4758	0.0447	0.2428	0.4758	0.4758	0.2864	0.1128
*R^2^*	0.9671	0.9790	0.9786	0.9388	0.9627	0.9786	0.9634	0.9730	0.9786	0.9786	0.9753	0.9735
Pseudo-second-order												
*k_2_* × 10^−3^ (g/mg·min)	1.334	1.680	2.111	7.918	3.353	2.111	0.320	0.962	2.111	2.111	1.403	0.568
*q_e,cal_* (mg/g)	502.04	766.49	798.59	264.45	488.40	798.59	968.63	905.77	798.59	798.59	498.22	467.75
*R^2^*	0.9859	0.9903	0.9909	0.9889	0.9907	0.9909	0.9958	0.9903	0.9909	0.9909	0.9848	0.9855
Elovich												
α	469.277	476.972	510.961	121.800	230.274	510.961	48.224	226.407	510.961	510.961	160.041	98.114
*β*	0.0081	0.0040	0.0038	0.0101	0.0054	0.0038	0.0028	0.0029	0.0038	0.0038	0.0059	0.0073
*R^2^*	0.9807	0.9500	0.9431	0.9393	0.9556	0.9431	0.9463	0.9561	0.9431	0.9431	0.9475	0.9396
Intraparticle diffusion												
*k_i_*_1_	170.21	138.16	138.98	42.55	109.87	138.98	53.83	118.58	138.98	138.98	73.25	62.15
R^2^	0.9246	0.9459	0.9246	0.9246	0.9246	0.9246	0.9246	0.9399	0.9246	0.9246	0.9246	0.9246
*k_i_*_2_	184.23	411.62	432.87	132.14	259.82	432.87	159.77	340.72	432.87	432.87	197.84	138.11
*R^2^*	0.9548	0.9910	0.9928	0.9937	0.9895	0.9928	0.9933	0.9930	0.9928	0.9928	0.9930	0.9843

**Table 3:** Modeling of breakthrough curves for the adsorption of lysozyme by using the Thomas, Bohart–Adams, BDST, and Yoon–Nelson models.

Models																			
Operating Parameters				Thomas				Bohart–Adams				Yoon–Nelson				BDST			
Z (μm)	pH	*C_o_* (mg/mL)	*F* (mL/min)	*q_eq_*	*q_eq_* *(exp)*	*R^2^*	*E* (%)	*N_o_*	*N_o_* *(exp)*	*R^2^*	*E* (%)	*t_0.5_*	*t_0.5_* *(exp)*	*R^2^*	*E* (%)	*N_o_*	*N_o_* *(exp)*	*R^2^*	*E* (%)
115	5	2	1	289.252	459.048	1.000	58.702	191.403	161.827	0.978	15.452	2.169	2.330	1.000	7.401	101.969	161.827	1.000	58.701
115	7	2	1	668.131	733.552	0.997	9.792	282.674	258.596	0.913	8.518	5.011	5.018	0.997	0.140	235.586	258.596	0.997	9.767
115	9	2	1	724.858	769.649	0.910	6.179	318.876	271.322	0.821	14.913	5.436	5.497	0.910	1.114	258.354	271.322	0.910	5.019
115	9	0.5	1	248.667	258.754	0.903	4.056	103.885	91.218	0.803	12.193	7.460	7.500	0.903	0.536	88.390	91.218	0.903	3.200
115	9	1	1	434.273	473.958	0.917	9.138	188.782	167.083	0.903	11.494	6.514	6.546	0.917	0.490	154.530	167.083	0.917	8.123
115	9	2	0.1	906.659	961.588	0.896	6.058	385.754	338.985	0.773	12.124	67.999	68.593	0.896	0.873	323.035	338.985	0.896	4.938
115	9	2	0.5	830.626	875.872	0.961	5.447	358.463	308.768	0.815	13.863	12.459	12.525	0.961	0.524	294.054	308.768	0.961	5.004
345	9	2	1	446.741	469.920	0.874	5.188	190.328	165.659	0.993	12.961	10.052	10.366	0.874	3.129	158.373	165.659	0.874	4.601
575	9	2	1	400.782	433.789	0.960	8.236	167.233	152.922	0.917	8.557	15.029	14.863	0.960	1.106	141.275	152.922	0.960	8.244

## Data Availability

Due to the nature of this research, participants in this study did not agree for their data to be shared publicly, so supporting data are not available.

## Nomenclature

*A*	Nanofiber membrane’s effective area (cm^2^)
*BV*	Bed volume
*C_0_*	Inlet lysozyme concentration (mg/mL)
*C_t_*	Concentration of lysozyme in effluent at time t (mg/L)
*DBC*	Dynamic binding capacity (mg/g)
*EBC*	Equilibrium binding capacity (mg/g)
*F*	Flow rate (mL/min)
*HMTZ*	Length of the mass transfer zone (μm)
*J*	Permeation flux (L/m^2^·h·bar)
*k_1_*	Pseudo-first-order kinetic rate constant (1/min)
*k_2_*	Pseudo-second-order kinetic rate constant (g/mg·min)
*k_BA_*	Adams–Bohart kinetic constant (mL/(mg·min))
*k_BDST_*	Rate constant for BDST model (mL/min g)
*k_i_*	Intraparticle diffusion rate constant (mg/g·min^0.5^)
*k_T_*	Constant for Thomas model (mL/min mg)
*k_YN_*	Rate constant for Yoon–Nelson model (1/min)
*MAER*	Membrane adsorber exhaustion rate (g/mL)
*MBU*	Membrane bed utilization (%)
*N_o_*	Saturation binding capacity of membrane bed (mg/mL)
*P*	Productivity (mg/g·min)
*q_1_*	Binding capacity of the membrane (mg/g)
*q_2_*	Binding capacity of the membrane (mg/g)
*q_t_*	Binding capacity at any given time t (mg/g)
*q_e_*	Equilibrium binding capacity (mg/g)
*q_e(exp)_*	Experimental equilibrium binding capacity (mg/g)
*Q_o_*	Binding capacity of membrane per unit bed volume (mg/mL)
*t*	Service time (min)
*t_b_*	Time required at 10% breakthrough (min)
*V*	Total volume of permeated solution (mL)
*V_b_*	10% breakthrough volume (mL)
*V_M_*	Membrane volume (mL)
*W_M_*	Weight of the membrane adsorber (g)
*Z*	Length of the membrane bed (cm)
*v*	Superficial (linear) velocity (cm/min)
τ	Residence time in the membrane (min)
α	Initial adsorption rate in the Elovich kinetic model (mg/g·min)
β	Constant related to the extent of surface coverage and activation energy for chemisorption in the Elovich kinetic model (g/mg)
ε	Porosity of the membrane
